# Health-Oriented Environmental Categories, Individual Health Environments, and the Concept of Environment in Public Health

**DOI:** 10.1007/s10728-023-00477-5

**Published:** 2024-01-29

**Authors:** Annette K. F. Malsch, Anton Killin, Marie I. Kaiser

**Affiliations:** 1 Faculty of Health Sciences, AG7 Environment and Health, Bielefeld University, Universitätsstraβe 25, 33501 Bielefeld, Germany; 2 Department of Philosophy, Bielefeld University, Bielefeld, Germany; 3 Joint Institute for Individualisation in a Changing Environment, University of Münster and Bielefeld University, Münster and Bielefeld, Germany

**Keywords:** Definition of environment, Environmental health determinants, Human ecology, Individualisation, Public health, Salutogenesis, Social environment

## Abstract

The term ‘environment’ is not uniformly defined in the public health sciences, which causes crucial inconsistencies in research, health policy, and practice. As we shall indicate, this is somewhat entangled with diverging pathogenic and salutogenic perspectives (research and policy priorities) concerning environmental health. We emphasise two distinct concepts of environment in use by the World Health Organisation. One significant way these concepts differ concerns whether the social environment is included. Divergence on this matter has profound consequences for the understanding of health and disease, for measures derived from that understanding targeting health promotion and disease prevention, and consequently, for epistemic structures and concept development in scientific practice. We hope to improve the given situation in public health by uncovering these differences and by developing a fruitful way of thinking about environment. Firstly, we side with the salutogenic conception of environment as a *health resource* (as well as a source of health risks). Secondly, we subdivide the concept of environment into four *health-oriented environmental categories* (viz., natural, built-material, socio-cultural, and psychosocial) and we link these with other theoretical notions proposed in the health sciences literature. Thirdly, we propose that in public health ‘environment’ should be understood as consisting of all extrinsic factors that influence or are influenced by the health, well-being, and development of an individual. Consequently, none of the four categories should be excluded from the concept of environment. We point out the practical relevance and fruitfulness of the conception of environment as a health source and frame this in causal terms, representing *individual health environments* as causal networks. Throughout, we side with the view that for the design of human health-promoting settings, increased attention and consideration of environmental resources of salutogenic potential is particularly pressing.

## Environment, Health, and the Public Health Sciences

The term ‘environment’ lacks a consistent definition across the public health sciences, including within environmental public health. As we’ll discuss, even the concepts of environment in use by the World Health Organisation (WHO) at the international level and by WHO Europe, defining the field of environmental health science, diverge distinctly. The concept of environment for WHO International includes only the natural, the built, and the working environment. This reflects a particular focus: concentrating on human health protection against direct pathological environmental risks. WHO Europe include additionally the social environment, both its direct and indirect effects on human health and well-being. This reflects the aim to implement environment-related human health promotion measures.^1^

It is important to note that these two different environment concepts have engendered distinct action areas for the public health sciences. The first concentrates on the environment as a source of health risks, and consequently on public health measures to prevent morbidity. On this line of reasoning, sustainable environmental management would be sufficient to solve environmental health problems and need not be an area of further action for public health practitioners. By additionally including the social environment, the second concept helps to better facilitate an additional research and action field for the public health sciences: *salutogenesis*, the study of health-promoting factors (contrasted with *pathogenesis*, the study of disease-causing factors), which in turn better facilitates applied scientific projects such as the design of health-promoting human living and working environments. It does so by expanding what counts as an environmental health determinant, to include additional kinds of determinants for which a salutogenic potential can be tested. The status of the salutogenic potential of the purely ‘physical’ environment (non-social, non-psychological environment) is controversial and under-studied compared to that of our social and psychological worlds [22], even though the salotugenic/pathogenic distinction is in principle orthogonal to the matter of the definition of environment.

We said above that an action area for the public health sciences concerns environmental salutogenic potential. This runs counter to a recent philosophical account of environmental public health, which, despite being somewhat expansive (admitting social, biological, chemical, and physical factors), only includes hazards—health risks—within its scope [20]. To develop salutogenic (health-promoting) human spaces, however, the environment should also be considered as a health resource [11, 54], rather than merely a source of risk. A priority for the public health sciences is then the evidence-based characterisation of environmental features as health promotion measures. German public health provides one example of this in practice. Since 2015, health promotion has been anchored in German legislation (SGBV §20a), and as a result, health promotion by environmental conditions is increasingly becoming the focus of attention of German public health research and care.^2^

The design of salutogenic living and working environments, especially in urban areas, is a challenge, but it also serves as a highly valuable instrument for tackling social inequality and injustice. After all, health promotion is defined as a process that should enable all people to have a higher degree of self-determination over their own health and thereby empower them to strengthen their health (WHO Ottawa Charter for Health Promotion 1986).^3^ This means that the design of health-promoting human environments must also be suitable for enabling each individual to engage in a personal process of development and maturation. The quality, diversity of use, aesthetics, atmosphere, safety, etc., of the physical environment all play significant roles in health-relevant personal development opportunities [23]. Moreover, acquiring, developing and conquering new skills of course takes place in an environment and strengthens, among other things, self-confidence, self-esteem, body awareness, sense of orientation and self-perception [23]. Hence, the salutogenic setting is a place where features of the environment, including (psycho-)social arrangements, jointly promote health in a collective sense, and where policies that value health are effective at all levels of society [38].

Thus the two different environment concepts of WHO have far-reaching consequences for research and practice in the public health sciences, for understanding health and disease, for measures derived from that understanding, and for disease prevention and health promotion.

Even so, the issue at stake is no mere semantic quibble. For example, a recent scientific exchange (concerning the assessment of the environmental burden of disease) serves to reveal disagreements and related problems arising from different environment concepts in human carcinogenesis research exemplarily. Prüss-Üstün et al. [41] estimates that 19% of all cancers are attributable to environmental causes, a figure criticised by Boffetta et al. [9] as an overestimation “of an order of magnitude” (p. 913). The disagreement can’t (or shouldn’t) be considered merely academic; as the former authors represent the World Health Organisation and the latter represent the International Agency of Research on Cancer, potential knock-on effects for policy and public health promotion may be at stake. Boffetta et al. outline various types of error and biases that may have resulted in the alleged overestimation, only for Prüss-Üstün and Corvalán [42] to come back with a clarification of their methods, scope, and importantly, a clarification of their concept of environment. Namely, “our estimate is not attributable to pollution alone, but to all environmental modifications in a broader sense” (p. 1849). They list pollution, sanitation-related hygiene, occupational environment (and exposures to risks therein), UV radiation, and features of the built-material human environment, all as environmental causes. Boffetta et al. are of course aware of the ambiguity of the term ‘environment’ and its wide uses, though their analysis turns on a narrower conception, according to which “environmental factors are restricted to air, water, soil and food pollutants, including physical pollutants such as sources of ionizing radiation” (p. 913).

In order to avoid confusion and potential misinterpretation in the future, Boffetta et al. run an eliminativist line: that the term ‘environment’ should be cut from the scientific discourse—that pollutants be called ‘pollutants’ and other external factors be called ‘non-genetic’. Saracci and Vineis [46] caution against the ‘environment’-eliminativist recommendation, and we follow suit. ‘Environment’ is a vital, if not indispensable term, so we believe it is better to be clear and transparent on its definition than ban its use. Indeed, public health research and practice relies on the definition of health determinants influencing human health, and the distinction between health determinants internal and external to an individual. The positive or negative health effects of the individual interacting with its ‘surrounding’ (modulated by genetic predispositions and non-genetic mediators, vectors and confounders resulting in multifactorial health effects) is crucial for the health sciences in general. Inconsistent definitions and, more importantly, lack of clarity and transparency, lead to an inadequate understanding of environment whether as a source of health risks or a resource for designing sustainable, health-promoting spaces. This is reflected in not only the differences of scope but also the normative differences between the leading environment-related public health research and policy frameworks (viz., *One Health*, *EcoHealth*, and *Planetary Health*; see, e.g., [32]). So, our article is in part a call for clarity and transparency with respect to the use of ‘environment’ in and across the public health sciences, lest it be tossed aside as a lexical tool for breeding misunderstandings.

The road ahead is as follows. Next, we introduce the prevailing understanding of environment in public health, with an emphasis on environmental health, by discussing the Ottawa Charter and two concepts of environment due to the World Health Organisation (section "Environment in public health discourse"). We side with the conception of environment as a salutogenic health resource as well as a source of health risks, and recognise that even the more preferable (by our lights) of the World Health Organisation’s concepts does not stretch far enough. This is followed by an analysis of seven environmental ‘spheres’ as suggested by Barton [4] and Barton and Grant [5], which we condense into four *health-oriented environmental categories* (HEC) (section "Human health-related environmental interactions"). In doing so, we subdivide the concept of environment into four categories (natural, built-material, socio-cultural, and psychosocial), and we link these categories with other theoretical notions proposed in the health sciences literature. We propose that in public heath, ‘environment’ should be understood as consisting of all extrinsic factors that causally relate to the health, well-being, and development of an individual—and not just extrinsic factors that pose health risks (cf. [20]). Consequently, none of the four categories should be excluded from the concept of environment.

We follow the ecological strategy of conceiving of an environment *indexically*, in relation to a focal individual. Indeed, we represent the health-related environment of an individual human as a causal network and provide a schematic example (section "Individual health environments"). This *individual health environment* (IHE) representation serves as both a clarification of our environment concept and a potential tool for causal modelling and hypothesis testing, differentiated enough to be applicable in the myriad interdisciplinary contexts of (environmental) public health science.

Finally, our "Conclusion" wraps things up. Throughout the article, our aims are not merely definitional, but also to ignite philosophical analysis more broadly of environment in the health sciences.

## Environment in Public Health Discourse

This section serves as an historical and conceptual backdrop. Herein we contextualise our objectives with reference to significant environment-related public health frameworks and concepts. We provide a philosophical analysis of the epistemic-conceptual practice of public health, with respect to notions of environment. As such, our analysis is concerned with implicit ‘understandings’ as well as concepts engineered for use in political discourse and the public health sciences. These are concepts that were intended to do work (e.g., impact public health policy) and guide both research and practice.

### The Ottawa Charter

The WHO Ottawa Charter for Health Promotion of 1986 has been immensely influential in public health research and practice [24]. Given that it aimed to describe the basic conditions and constituent features of health—to which any improvements in health must be, one presumes, inevitably linked—it also laid the foundation for environment-related human health as a bona fide research agenda of the public health sciences. This is because (in addition to socio-cultural values such as social justice and equal opportunity) environmental conditions such as a stable ecosystem, adequate housing conditions, and sustainable use of existing natural resources were described as not merely desirable but ‘basic prerequisites’ for health and wellbeing in the Charter. It stated that “inextricable links between people and their environment constitute the basis for a socioecological approach to health”, and explicitly named environmental factors as a target of health promotion action. By doing so, the Ottawa Charter clearly pronounced the inseparable connection of social and environmental health determinants, calling for a more holistic approach to understanding health one year before the Brundland Report of 1987 for sustainable development. Among the commitments to health promotion to which the conference participants pledged is “to counteract the pressures towards harmful products, resource depletion, unhealthy living conditions and environments, and bad nutrition; and to focus attention on public health issues such as pollution, occupational hazards, housing and settlements”. Ecology was named as an essential research area for “developing strategies for health promotion” on the same level as social justice. All this is linked to the tasks of environment-related health protection, by calling for the systematic recording of the health consequences of our rapidly changing environment as a basic requirement for health promotion and formulating it as a core social task. And needless to say, in the intervening 38 years, many health science researchers and practitioners have made admirable progress. While the main focus of research emphasises health protection via the management of anthropogenic environmental pollution (quite understandably), there has unfortunately been much less work emphasising and resolving the challenges of the kind of dynamic, interconnected environmental approach claimed by the Ottawa Charter.

The Ottawa Charter, then, proffers an understanding of the environment that aims for a sustainable way of life—an understanding that could function as a socio-ecological ‘nucleus’ for researchers developing health-promoting strategies. Accordingly, people are held responsible as designers of their immediate environment, as human persons who think about the health of the living environment and influence it decisively by their individual and collective activities.

Next, we describe how the understanding of environment in the Ottawa Charter has been taken up and modified in global health policy. For this, we contrast WHO International and WHO Europe conceptions.

### WHO International

By 2014 WHO International defined environmental health as encompassing “all the physical, chemical, and biological factors external to a person, and all the related factors impacting behaviours… targeted towards preventing disease and creating health-supportive environments (including clean air and water, healthy workplaces, safe houses, community spaces and roads and managing climate change). This definition excludes behaviour not related to environment, as well as behaviour related to the social and cultural environment, and genetics” (WHO, cited in [8], p. 364).

The last sentence of the definition makes clear the intentional separation of ‘environmental’ conditions from socio-cultural interactions and human structures. The purported justification for this is along pragmatic grounds: here we have a practical and manageable definition of environmental health, focusing as it does on specific features of the world that undeniably influence health and can be at least in principle changed through environmental management.

By 2020, however, developments were afoot. The WHO Global Strategy on Health, Environment and Climate Change (2020) no longer expressed such a hard-line separation. No longer were healthy workplaces conceived as a mere goal or target. Rather, the scope of environmental health was explicitly extended to include the work-related environment. This was a step in the right direction, to be sure, for work environments from deep mines to high-rise office spaces can of course (and in some cases, drastically) influence an individual’s health. Nevertheless, the approach to environmental health remained limited to a pathogenic perspective, focusing on the environment as a source/origin of *risk factors*: “Environmental risks to health, in the framework of this strategy, are defined as all the environmental physical, chemical, biological and work-related factors external to a person, and all related behaviours. It focuses especially on the part of the environment that can reasonably be modified” (p. 1).^4^ The Strategy sets, as its idealised goal, “a world in which sustainable development has eliminated the almost one quarter of the disease burden caused by unhealthy environments… and which manages environmental risks to health” (p. 4).

According to the Strategy, for a safe, supportive and equitable environment, people must first and foremost change the way they “live, work, produce, consume, and govern” (p. 4). Air pollution, unsafely managed water, and unsafe workplaces are emphasised as causes of millions of preventable deaths every year; climate change and loss of biodiversity are also identified as risks to health, especially to island and low-lying populations and the least economically developed countries. Of course, none of this is surprising. And while the Strategy is not wrong that specific changes will likely lead to improvements in health (and reductions in preventable deaths), and while its objectives mention the promotion of healthy choices, its otherwise admirable vision falls short of a broader contextualisation of environment and health promotion. This is a missed opportunity, not only because the actual causes of the described health-related environmental problems are primarily attributable to global social inequality or environmental injustice. Rather, the framework notably lacks consideration of the dynamic and close-knit interrelationships between people and the environment from a salutogenic perspective. In this way, the Strategy departs from the spirit of the Ottawa Charter.

### WHO Europe

The WHO Regional Office for Europe apparently took a different definitional pathway. The European Charter on Environment and Health (1989) draws on a broader understanding of ‘environmental health’. The Charter defined it to encompass “both the direct pathological effects of chemicals, radiation and some biological agents and the effects (often indirect) on health and well-being of the broad physical, psychosocial, social and aesthetic environment, which includes housing, urban development, land use and transport” (p. 18).^5^ Although at that time no concrete measures for environment-related health promotion were formulated, doing so was recognised as a priority for future progress: “Health promotion should be added to health protection as to induce the adoption of healthy lifestyles in a clean and harmonious environment” (p. 13). This was based on the realisation that myriad environmental factors influence an individual’s health positively or negatively.

Following two significant WHO Europe developments—the Concern for Europe’s Tomorrow (1994)^6^ and the Environmental Health Action Plan for Europe (1994)^7^—national and regional action plans, in time, emerged. From this action, on the national level in Germany (for example) currently only the Master Plan for Environment and Health (2016) of North Rhine-Westphalia is left. This plan calls for the creation and design of health-promoting environments, especially with regard to environmental justice. Reducing social inequality in relation to risks and opportunities afforded by the environment is conceptualised as a cross-sectoral issue for which a joint commitment of all policy areas is a priority. In this manner, the Plan implicates the fact that environment-related conceptions of health promotion must extend to social, economic, legal and political structures, or fail to capture all that is salient. Only then can the full range of burdens on human health and key opportunities for improvement be identified and taken into account in a cross-sectoral, inclusive and systematic environmental policy.

### Zooming Out

Divergent understandings and perspectives of environment such as those described in the preceding sections have inevitable consequences for research and practice. In much public health research, the physical environment is recorded as a *contextual* factor only, or else considered merely as the (external) physical space/location where health promotion takes place—even though that health promotion occurs mainly on a psychosocial and socio-cultural level (by way of behaviour-oriented measures, for the most part). As a result, the nature and design of the environment, and the interaction of people with it, are considered to be of ancillary importance, if considered at all. Generally, the main environmental focus is on risks and sources of pollution/disease, whereas environmental resources of salutogenic potential are much less in focus [22]. That said, more recently, there has been a spike of interest in the health-promoting effects of nature in urban areas (e.g., [10, 12, 19, 56]), driven by increased interest generally in urbanisation and climate change. A typical focus is on green (‘urban nature’) and blue (‘urban water’) infrastructure as a health resource [29, 34, 52, 56]. How closely human health is linked to environmental conditions is also becoming increasingly clear due to sustainability research on the conflicts and synergies between the various goals of the United Nations 2030 Agenda (of 2015). For example, the 2030 Agenda’s ‘Sustainable Development Goal 3’ on health and well-being is closely linked to all other goals and has been proposed as a promising indicator of progress for the 2030 Agenda overall [39].

Even so, only if environmental features (still to be characterised) are understood as a health resource (instead of only pathogenically as a health *risk*)—and hence identified and evaluated as such—can their possible salutogenic influence on health be properly examined and used to design health-promoting human environments.

The remainder of this article attempts two main tasks. First, we propose a framework of reference for the concept of environment in public health research and practice (the ‘health-oriented environmental categories’, or HEC).^8^ Second, we develop a theoretical perspective of ‘individual health environments’ (IHE) building on an ecological, causal-network conception of environment (and incorporating HEC), to illustrate our conception of environment and to provide a tool for representing causal relations between individuals and environmental health determinants (both positive and negative). We turn now to the first of these two tasks.

## Human Health-Related Environmental Interactions

As described in section "Environment in public health discourse", which external features get to be included and indeed characterised as environmental health determinants depends in large part on the environment concept at hand. One crucial aspect of the presented differences in environment concepts concerns the assignment of *social* health determinants as *environment*. Even now, debates persist about the importance and indeed the very concept of environmental factors in relation to social factors (see section "Environment, health, and the public health sciences"). In our view, this seems not to be productive, because it sets the two against each other instead of concentrating on their interaction. Both are interrelated and jointly responsible for health inequalities and injustices [6, 7, 37].

However, the ‘rainbow model’ of Dahlgren and Whitehead [14] is the most broadly accepted model of environment in the public health sciences, used to illustrate the main influences on health, such as employment/work environment, education, water and sanitation, housing, agriculture and food production, individual lifestyle factors and community networks.^9^ Another model is the Diderichsen and Hallqvist framework [15, 17, 18] which concentrates on social health determinants, while relegating (other) environmental determinants to risk factors only. The ‘material conditions’ (*sensu* [14]) are not identified as an environmental health task. For these reasons, we set it aside. In our view, a great step forward was the conceptual model of Schultz and Northridge [49], which examines the relationship between social inequalities, the natural and built environment, and the social context, by contrasting social and (other) environmental health-related factors.

However, at this point, we note that current models of health determinants were developed with specific purposes in mind and therefore reflect different (sub-)disciplinary perspectives and approaches to environmental/social factors. Depending on the model chosen, empirical studies will inevitably apply different categories accordingly. Hence, an overall environment concept without compromising narrowly interwoven social factors as aimed at in this paper would contribute to the field, increasing the comparability for the interdisciplinary domain that is public health research and action.

### Health-Oriented Environmental Categories (HEC)

We take as an initial basis for our environment concept the integrative and—due to its environmental layers—differentiated ‘health map’ of Barton and Grant [5] for several reasons. First, Barton and Grant’s map intentionally integrates antecedents in the health sciences literature. It references the rainbow model of Dahlgren and Whitehead for contextualising the social environment and it follows the conceptual approaches of urban sustainable development as well as an integrative human ecological perspective. By doing so, it coheres with the Ottawa Charter and the WHO EU approach to environmental health (see section "Environment in public health discourse"). Moreover, as a model from urban health research it provides a detailed systemic description of the health-relevant interactions between humans and their surrounding environment [4]. As a conceptual heuristic, the health map can be applied to single individuals as well as groups of people. Hence, the health map is well suited as a starting point to propose a conceptual environmental framework for public health discourse as well as the health sciences generally.

**Fig. 1:**
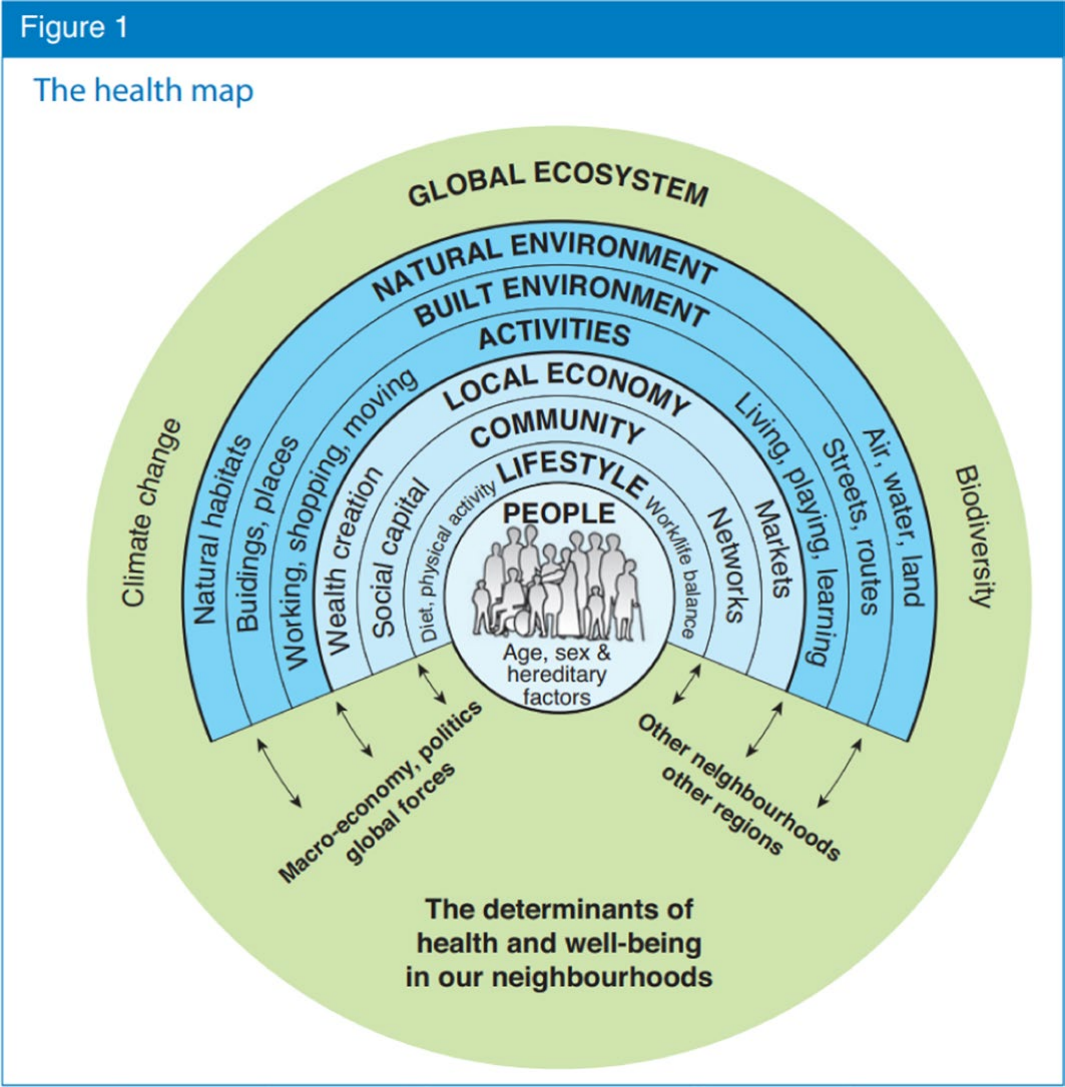
The health map. Illustrates the interactions between people and their environment (figure taken from [5], p. 252). Figure reproduced under STM Permissions Guidelines

The health map (Fig. 1) has served as a basis for the development of systematic and sustainable urban district development. It represents an example of the ecosystem of a human settlement, nested as it is within the global ecosystem, and which can be applied to ‘settlements’ broadly (e.g., village, neighbourhood, city, metropolitan region, etc.). Barton and Grant [5] outline the interactions between human health and environment of the seven spheres, and this explicitly includes human activities/behaviours, in turn modulated by such factors as human individuals’ genetic predispositions. The sum of human interactions with the different environmental spheres interacts with climate and biodiversity.

Table 1 tabulates this map and links its ‘spheres’ to our HEC (natural, built-material, socio-cultural, and psychosocial environment; see below) and other theoretical notions from the literature by visually relating the corresponding rows of the various columns.

**Table 1:**
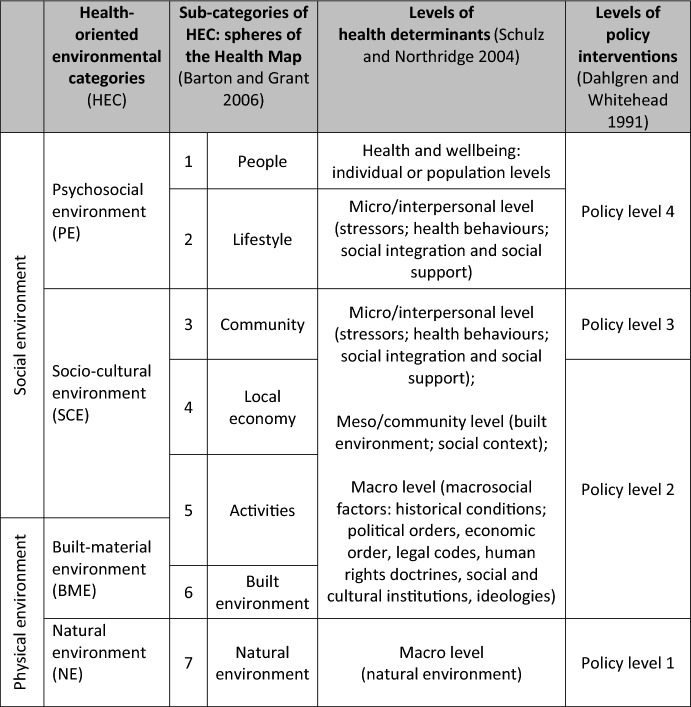
Health-oriented environmental categories (HEC) and related terminology

There are four health-related environmental categories (HEC) and the corresponding environmental spheres of the health map [7], the corresponding levels of social analysis connecting social and environmental health determinants by Schulz and Northridge [55], and levels of policy interventions by Dahlgren and Whitehead [17]

Each health map ‘sphere’ represents both a process and a state of affairs: each sphere’s qualities—whether societal, economic, ecological, etc.—can be measured at a given point in time, at least in principle, bearing in mind that things are constantly evolving, influencing the other spheres and the encompassing circle, and generating feedback. Given that these spheres are interacting, dynamic, and evolving, we should emphasise, as others have, that “the environment of an organism is neither definitive nor static, but is formed with the development and activities of the organism” [40], p. 161.

By considering the contexualisation of the seven spheres, from central sphere 1 (‘people’) to outer sphere 7 (‘natural environment’) with regard to their characteristics, significance, theoretical context and analytical value, we provide theoretical grounds for distinguishing four health-oriented environmental categories (HEC). We refer to these as the natural, built-material, socio-cultural, and psychosocial environment. While these may be familiar terms, incorporating external psychosocial features into ‘environment’ is not a familiar move; moreover, we take our analysis of the four categories to be novel: these categories bracket the seven spheres of the health map into easy-to-use environmental main units, with the spheres still serving as sub-categories. And they are a helpful orientation in interfacing with several other public health frameworks (see Table 1), and characterising environmental health determinants in an individual health environment (section "Individual health environments").

The following sections comprise a detailed description and justification of the four HEC. Where necessary, we go beyond the concept of the health map to clarify the differences between the HEC categories as well as to add the interpersonal level as psychosocial environment. We begin with the natural and built-material environment categories.

### Natural Environment and Built-Material Environment

*Natural environments* (sphere 7) consist of physical substrates and forces as well as individual organisms (plants, animals, fungi, bacteria and viruses), comprising many different kinds of ecosystems, from micro-ecosystems (e.g., a pond in urban nature) to macro-ecosystems (e.g., a nature reserve), all of which are interconnected within the global ecosystem. Human settlements are embedded in the surrounding landscape and rely on it for the provisioning of essential resources such as clean air, land, water, building materials, and energy. Even if such landscapes can no longer be described as legitimately ‘natural’ in the sense of pristine, original, or unmodified by human activity, residents appreciate them for a wide variety of reasons: for a landscape’s intrinsic value (e.g., as homeland, its cultural associations and identity), its habitats for wildlife, and its economic and recreational affordances. Moreover, even if some urban areas are richer in species diversity than conventional agricultural land, a pragmatic and clear enough demarcation can be drawn between ‘natural’ and ‘built-material’ environment, the next category. Of course, humans and their hominin ancestors have developed over the course of evolution in natural environments, but have also directly shaped their environments [30]. So, the distinction will admit of fuzzy/borderline cases. Still, it admits of very many paradigm cases too. The buildings, streets, vehicles, artificial materials and products as well as artefacts between settlements and inside a settlement’s walls belong to the built-material environment, as do the walls. The river flowing through the settlement, the parks and recreation areas and trees along the streets, the mountains and woodlands yonder, the birds in the sky and insects in the meadow, belong to the natural environment.

The human *built-material environment* (sphere 6), then, is created/modified by humans, through the use of raw materials and the development of new artificial materials, consisting of purpose-built places (buildings, town squares, railway stations, etc.), between which there are mobility structures for movement/transportation (paths, roads, canals).^10^ We include artificial and augmented reality in this category as well, even if mixed and virtual environments have the potential to represent a unique category in future. The needs and the type of usage (sphere 5, *activities*) defines the construction of a human built-material space, falling under both the built-material and the socio-cultural domains. Moreover, human interpersonal activities characterise personal *lifestyle* (sphere 2): human activities, in interaction with various built-material elements, like working, eating, shopping, playing, exercising and communicating pose requirements for the construction of spatial and, in the digital age, virtual structures. By the provisioning of built-material structures, people are able to facilitate service activities like wastewater treatment and energy generation. Different human activities utilise—and wear through—myriad anthropogenic tools, instruments, and products mainly made of artificial material; this results in billions of substances/particles accumulating in environmental media (including plants, animals, and other humans). The availability, quality, and safety of the built-material environment with which we interact on a daily basis (in our homes, offices, and school buildings) have significant direct and indirect pathogenic effects on health. However, the scope and potential of salutogenic resources of natural and built-material environments waits largely to be discovered for shaping health-promoting living conditions for the better.

Together, the natural and built-material environments comprise what is sometimes referred to as the ‘physical environment’ and is contrasted with the ‘social environment’ (this nomenclature is intended in an ontologically neutral way; it does not entail that socio-cultural and psychosocial features are nonphysical). We turn next to the socio-cultural environment.

### Socio-Cultural Environment

The ‘socio-cultural environment’ consists of many social and cultural structures, within and with which various forms of human behaviour take place (sphere 5, *activities*). Income (or, more broadly, ‘financial health’) is a significant social determinant of health (e.g., [55]), so the general structure and dynamics of the local economy and the extent to which it creates employment opportunities for different groups of the population is highly relevant for health (sphere 4, *local economy*). When spatial structures of the built-material environment such as post offices, banks, health authorities or hospitals close local branches, they pass on financial and health costs to local customer groups. Within these systems, people (e.g., as stakeholders, employees, etc.) take on representative roles for particular systems and represent its rules and values. This is how interpersonal interactions (or activities) create culturally-shaped normative social rules and structures as well as social networks and communities (sphere 3, *community*) in which the provisioning of social structures for integration in terms of environmental justice, participation, and empowerment also plays an important role in health. The local community and its structures can be highlighted as crucial for social inclusion (such as with regards to schools, shopping opportunities, pubs, sports and games, political gatherings, community meetings, etc.) and thereby for individual health measures.

In summary, human socio-cultural environments are those anthropogenic and culture-oriented components of environments that comprise all collective structures and norms established (economic, political, social, legal, etc.), indeed which include human activities insofar as they create interactivities/interactions involving more than one human individual. And it is through this slice of the environment that framework conditions are created, in which and with which behaviour-oriented health promotion and prevention can take place, tasks of the public health sciences.

### Psychosocial Environment

In constant interaction with the various environmental conditions described above, *people* (sphere 1)—individually and collectively—determine their *lifestyle* (sphere 2) through their behaviours, based in part on their personal perception and intrinsic evaluation of their experience. Views about diet, exercise, smoking, gaming, and so on, are all lifestyle factors, influencing the activities that people engage in. New lifestyle changes—intentional or otherwise—can influence individuals in a range of ways (e.g., be empowering and healthy, or undermining and cause stress). Moreover, group-related experience of the environment (whether stratified according to age, gender, migration background, functional limitations, etc.) additionally lead to different perceptions and range of uses of the environment with correspondingly divergent health outcomes. Further aspects of individual behavioural choices, hereditary factors, epigenetics, as well as individual predispositions and vulnerabilities may also have decisive influence on environmental interactions. And by doing so, humans determine, change and shape their environmental conditions, mutually influencing each other: this too can take various forms, and be empowering, stressful, or otherwise. Hence, we claim that people are also features of each other’s environments in a very real sense. The mere presence of a particular individual may affect the well-being of another person as a stressor or resource; that is, as a health determinant. For example, it has been well established that people can easily, even if unintentionally, influence the mood and behaviours of others [13, 16, 51]. We humans are adept ‘mind-readers’ [27]—and our beliefs about others’ beliefs also influence our moods and behaviours. So the mental states of other humans too are implicated in an individual’s (psychosocial) environment.

The general approach here is supported by Rauthmann’s person-environment relations model [43]. Although it does not distinguish the social from the physical environment, this model identifies four person-environment interactions that influence personality: *interactions* (person and environmental variables moderate their mutual effects on outcome), *correlations* (person and environmental variables are simultaneously associated), *fits* (person and environment variables fit each other), and *transactions* (person and environment variables influence each other over time). In so doing, the model is able to specify various relationships and important impact pathways between person and environment as well as the resulting outcome variables.

### Applying HEC: Why and How

Altogether, HEC stratify the overall living/working environment of an individual (or group) (see Table 1). As described above, human interactions with HEC are complex and their evidence-based analyses are a demanding challenge for public health scientists. And since the public health sciences comprise a multidisciplinary research cluster, the current state of play reflects the on-going development of conceptual and methodological approaches within its sub-disciplines [47]. For example, three decades ago, when public health was established at universities in Germany, on the one hand the discipline was aligned with international, particularly Anglo-American models, and on the other hand, it was conceptualised in reviving aspects of the tradition of social hygiene [47]. Public health was conceived as an ensemble of individual scientific disciplines, ones directed towards a common subject area, that is, the improvement of the health of the population through disease prevention and health promotion [28]. The basic intention was for public health to gradually transform into a more unified, though interdisciplinary structure, with some hoping to see it develop into a new form of cooperation under a salutogenic umbrella [50]. All of this would require overcoming three central weaknesses of the discipline: the great distance between real-world health practices and university-based research, the rift between the social and natural sciences, and above all, the fact that the natural scientists, medics and social scientists working in public health had no common paradigm [3]. The difficulties in cooperation between the humanities and the natural sciences are not limited to public health. Typically, for the natural scientist, interdisciplinarity means renouncing effectiveness and explanatory/empirical precision, as well as the introduction of inhibitions; for the humanities scholar, it means ignoring critical contexts and bigger-picture philosophical concerns [53]. Even the political epidemiology that is very active and influential in the UK is characterised by a widespread absence of theory [47]. In this respect, it is perhaps obvious to consider the prospects that an integrative theory of the health sciences and a common set of methods might bring [44], [48]. A unified, *interdisciplinary* (not merely multidisciplinary) determination of (environmental) health determinants can only succeed if the scientific reference disciplines of the natural and social sciences can find themselves using a common language with a common standard. We suggest HEC to be such a conceptual tool for studies collecting environmental data (replacing disciplinary-specific terminology, e.g., ‘context factors’) as well as environment-related behavioural evaluations and interventions. This would enable a direct comparability of detected environmental health determinants from studies with quite different scientific paradigms. The examination and application of the four environmental categories and its seven sub-categories is intended to lead to a clearly differentiated, but comparable, easy-to-use approach to the human living environment.

Health-relevant human behaviour is one of the main research areas in the public health sciences (as in psychology, sociology, social work, pedagogics, etc.). Dividing the social context of an individual into two easily distinguishable environments, namely ‘psychosocial environment’ for interpersonal interaction and ‘socio-cultural environment’ for societal structures and norms is a theoretically and conceptually fruitful move. It facilitates and specifies the analysis and characterisation of health determinants (as well as their influence on each other). Social interpersonal interaction involves navigating rules and norms; the socio-cultural category covers implementation at the societal level including the creation of the structures in which the psychosocial is shaped by individuals.

Additionally, HEC allows the objective and subjective environmental perception of individuals to be surveyed and described in a much more differentiated way. For example, all aspects of health-relevant social interaction (psychosocial environment) could be examined in the context of subjectively perceived narratives or norms and externally imposed structures and rules (socio-cultural environment). This social context in turn takes place in a physical setting, which divides into two categories (the natural and built-material). Altogether, the four environmental categories stratify the overall living environment of human individuals and groups.

Since we are but at the beginning, there is more work to be done to refine and finesse HEC into a precise conceptual guide for the health sciences. And moreover, we invite other disciplines to assess our environment concept for practicability too. We propose that HEC offers a fruitful conceptual framework for a unified understanding of environment for inter- and transdisciplinary research, and for action on sustainable, eco-social, health-promoting transformations of our living spaces in the spirit of the Ottawa Charter.

## Individual Health Environments (IHE)

It is useful to represent the environment (as characterised in terms of HEC above) of a focal individual as a *causal network*, encompassing features external to a focal individual, of any environmental category, that effects their health, well-being and development.

But first, we recapitulate the core elements of our conception of environment and our philosophical assumptions. Our conception of environment stands in the salutogenic tradition of understanding environment as not only a source of health risks, but also a health resource [2]. For this reason, for our purposes, environmental categories should be ‘health-oriented’. From this we distinguished, above, four categories of environment: natural, built-material, socio-cultural, and psychosocial, and we discussed their health-relatedness. In this section, we add to this that we should conceptualise health-oriented environments indexically, on the individual level, including all features that are causally related to a focal individual’s health, well-being and development. To this end, causal networks are fruitful representations of an individual’s health environment.

We advocate an individual level conception because individuals differ (in their behaviour, personality, preferences, social interactions, environmental relations, and so on). Hence, the external health determinants of individuals are also different. Even in a single city, not all areas are equal in terms of exposure to pollutants, road noise, access to supermarkets, green/blue spaces like parks and rivers, and so on, again influencing different individuals’ health differently. This conception thus takes individualisation seriously (see also, e.g., [1]). Of course, scientific public health generally requires taking a *population*-level perspective on health determinants. But this is not incompatible with our approach. In population-level thinking, any description of a population is a statistical summary not intended to be applicable to any particular individual [36]. Even so, (evidence-based) population-level claims are typically licenced by statistically robust individual-level research claims (such as those vindicated by randomised control trials for experimental interventions).^11^ Our framework follows this rationale. By representing an environment in relation to a focal individual, we do not deny the importance of individual- and population-level interaction in health causation.

A focal individual’s environment, then, encompasses their psychosocial, socio-cultural, built-material, and natural environments (Table 1). At this point, it is important to reiterate that individual humans interact in and across groups, cultivating a common sense of how to interact with their environment. Yet one may ask, what does an individual’s health-oriented environment—features of the world external to the focal individual, characterisable by way of HEC—*look like*? That is, how should one conceive of the health-related environment of an individual person? This is a pertinent question as it is a longstanding strategy of ecology (among other disciplines) to define environment in terms of the features of a focal individual’s external surroundings (e.g., [26, 33, 35]). Here we apply this strategy to the health-relevant features of the external surroundings of a focal individual and offer an intuitive way to conceive of an individual health environment as a causal network. This we believe will facilitate the development of further formal modelling and hypothesis testing in research and practice.

We call the health-oriented subset of a focal individual’s full external environment—that which contributes, whether positively or negatively, to their health, development or wellbeing—an *individual health environment*. This is not a subjective demarcation, but an *indexically structured* one [31]. Just as there is no single fact of the matter about what the ‘time’ is (but rather, the time-at-Miami or time-at-Berlin, etc.), indexically structured composition of environment is objective (though may include subjectively experienced features). Within and across populations, individuals differ in their individual health environments: road traffic can be ignored completely by some people; for others, it triggers stress as an annoying background noise. An individual’s health environment can also change over time: road traffic might cause a person stress from middle age, but not have been problematic during their youth. Different features of the environment affect the health, well-being and development of individuals differentially; these differences can be more or less stable over time, consistent over context. This is a feature of our account: it takes individualisation seriously. Individual health environments draw attention to the interwoven nature of causal nodes and relations underpinning a focal individual’s health.

Causal relations/interactions are central to understanding and individuating individualised environments, and causal networks are a fruitful way of representing such environments. On such a representation, individual health environments “unfurl from a specific point of reference” [31], p. 503. In our case, this point of reference is a focal individual. Environmental features qua health determinants are thus conceived as nodes in a causal network, relating to the focal individual, other individuals, and each other, as the case may be. These features will inevitably span the range of the psychosocial, socio-cultural, built-material, and natural environment. The concept then provides a strategy for guiding causal modelling: “Once we identify which causal relations are relevant, we map where these causal actors are distributed” [31], p. 514—that is, the causal nodes that comprise the network—providing a guide to visual representation of the environmental health determinants with respect to a focal individual.^12^

The precise notion of ‘individual’ here is left open and can be operationalised differently in different research contexts. For example, the focal individual could be an individual organism (e.g., a human), a holobiont (e.g., an individual human plus their communities of microbiota; see [21]), or an individualised group (e.g., of humans). In the following we take an individual human as our (hypothetical) focal individual.

**Fig. 2:**
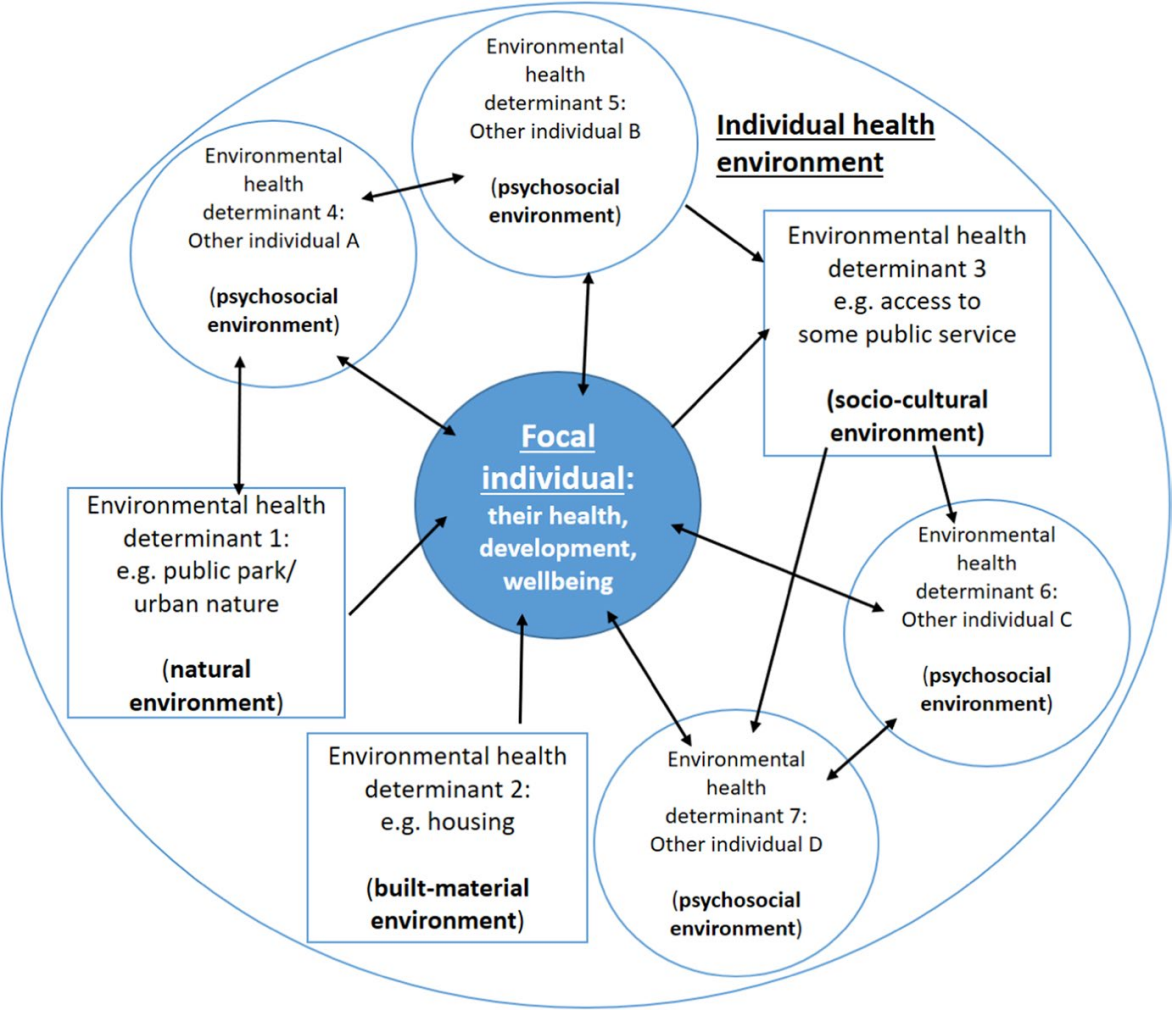
Individual health environment. A focal individual, dark inner circle, and their individual health environment (IHE), outer ring (a schema—comprising only four other individuals and three additional environmental health determinants). Illustrates a hypothesis of the causal relations between an individual and the environmental health determinants that comprise their IHE. Arrowheads represent direction of causation, which is bidirectional in the case of bidirectional arrows

A schematic representation (Fig. 2) serves as a much-simplified hypothetical example, purely for explanatory purposes. It expresses the concept of environment as a causal network, and displays causal relations between health determinants and a focal individual visually which may be used as a model for testing hypothesises about the causal extent and causal character of those relations.

According to Fig. 2, our focal individual is embedded in a causal network of (for simplicity’s sake) only *four* other individuals (psychosocial environment) and *three* additional environmental heath determinants, one each due to the natural, built-material, and socio-cultural environment and labelled ‘Environmental health determinant’ 1 through 3, respectively. The other individuals are labelled ‘Environmental health determinant’ 4 through 7, and are placed in circles rather than boxes (which are used for the other health determinants) for visual convenience and to reflect the fact that, like the focal individual, they are individual humans. This is not intended to suggest a different priority or ontological status with respect to the psychosocial environment. The overall circle, excluding the focal individual, though including the causal relations between the focal individual and the causal nodes, comprises an individual health environment (IHE).

The figure is to be interpreted as follows: assume it represents a hypothesis about the focal individual and how they stand in relation to external features that causally influence or are influenced by that focal individual’s health, well-being, and development. All of the other individuals in the network (determinants 4-7) influence the health of the focal individual, and the focal individual likewise influences their health (represented by the bi-directional causal arrows), which expresses the interpersonal dynamic of psychosocial environments. Individuals A and B also influence one another’s health (psychosocial environment), and the same goes for C and D. A and B do not directly influence C and D, or vice versa, but do so via indirect pathways. Individual B and the focal individual do something to influence the access of some public service (some feature of the socio-cultural environment—environmental health determinant 3) of individuals C and D, but are not directly influenced by that feature themselves (and nor is individual A). Of course, individuals A, B and the focal individual may however be indirectly effected, as a result of the influence that C and D have on the focal individual. Individual A and the focal individual are both influenced by a public park (natural environment—environmental health determinant 1), though only A does something to influence that determinant. Finally, the focal individual is influenced by some feature of the built-material environment (e.g., an aspect of their housing situation, perhaps poor ventilation—environmental health determinant 2).

Of course, measuring the actual impact of a single environmental factor upon an individual is elaborate. Of several factors, it is very demanding. And to include modulating factors as well as the interaction of multiple causally related factors, it is nearly impossible. The individual health environment as a concept provides only a guide, but one which enables public health scientists to *test*, by interventionist means, hypothesised single cause-effect relationships as well as multiple interrelated causal relations between (potentially significant) environmental health determinants and focal individuals.

Our purpose here is to provide IHE as a concept of environment and show how it can look as a research tool, incorporating HEC. We believe that the development of further uses for it in public health research and practice is a promising prospect: a priority for future research.^13^

## Conclusion

Public health is significantly impacted by concepts of environment-related human health. However, and in our view, unfortunately, the ‘environment’ part of that phrase is under-defined and under-analysed. Discussing and developing a conceptual framework for environment should be given a high priority. Importantly, we have drawn attention to the lack of a unified concept of environment in the public health sciences. This has knock-on effects, impacting research, practice, policy, and public understanding. In the tradition of understanding environment as a salutogenic resource, we argued that public health’s environment concept should be ‘health-oriented’ in a broad sense. We distinguished four categories of environment: natural, built-material, socio-cultural, and psychosocial, and discussed their health-relatedness. We proposed that public health’s environment concept should range over all extrinsic factors that influence the well-being, health and development of a focal *individual*, whether these determinants are due to the natural, built-material, socio-cultural, or psychosocial environment—all legitimate categories of environment. We conceptualise health-oriented environments indexically, encompassing all features causally-related to a focal individual’s health, well-being and development. To this end, we argued that causal networks are fruitful representations of an individual’s health environment.

## Footnotes

1 Henceforth, ‘health’ continues to refer to human health unless otherwise specified.

2 See, for example, the German BZgA (Federal Centre for Health Education) online encyclopaedia and articles therein, https://leitbegriffe.bzga.de/alphabetisches-verzeichnis/ (Accessed 23 January 2024).

3 The Ottawa Charter may be accessed at https://intranet.euro.who.int/__data/assets/pdf_file/0004/129532/Ottawa_Charter.pdf (Accessed 23 January 2024).

4 The Global Strategy on Health, Environment and Climate Change may be accessed at https://apps.who.int/gb/ebwha/pdf_files/WHA72/A72_15-en.pdf (Accessed 23 January 2024).

5 The European Charter on Environment and Health may be accessed at https://apps.who.int/iris/bitstream/handle/10665/272715/9789289011266-eng.pdf (Accessed 23 January 2024).

6 Summary of the Concern for Europe’s Tomorrow may be accessed at https://iris.who.int/bitstream/handle/10665/272870/9789289013178-eng.pdf (Accessed 23 January 2024).

7 The Action Plan may be accessed via https://www.eea.europa.eu/policy-documents/environmental-health-action-plan-for (Accessed 23 January 2024).

8 Although we argue for the fruitfulness of our framework, it is not meant to replace other specific environment concepts useful for other research agendas: in studying how cells communicate, for example, cell biologists speak of the extra-cellular environment, a narrower concept. How best to conceive the environment depends, in part, on the goals of the researcher/discipline. Where public health is concerned, we advocate for an expansive concept. We thank an anonymous referee for probing us on this issue.

9 As of January 2024, Dahlgren and Whitehead [14] has been cited over 4400 times according to Google Scholar.

10 We focus on human environments but a built-material environment need not be a human one: beavers and their dams as well as birds and their nests provide the classic non-human examples [25].

11 Consider the ecological viewpoint: “There are no phenomena operationally significant to such aggregates of organisms as species, stands, communities, associations, or floras, except as summations or as logical products of phenomena operationally significant to the included or associated individuals… It is easy to translate to aggregate phenomena after the environmental relations of the individuals are established and understood” [35], p. 331.

12 For an introduction to causal network graph analysis, though in a different (yet still interdisciplinary) context, see, e.g., Roberts et al. [45]. Lean [31] focuses on the case of ecological communities. For the use of causal graphs in a public health context—modelling childhood obesity—see Zhu et al. [57].

13 Detailed discussion of such uses and applications must be left for another time. Here we can only be brief. With IHE, researchers have a well suited tool for mapping environmental health determinants of whatever practical or research setting is at hand; with this tool, factors can be listed, categorised, prioritised—just once, or via changes over time. The latter would be suitable to map dynamics of environmental health determinants in changing environments. The factors may be measured with quantitative as well as qualitative (mixed) methods.
